# Evaluation of Germplasm Resistance in Several Soybean Accessions Against Soybean *Fusarium* Root Rot in Harbin, Heilongjiang Province, China

**DOI:** 10.3390/plants15030379

**Published:** 2026-01-26

**Authors:** Xue Qu, Sobhi F. Lamlom, Guangqing Ren, Yuxin Sang, Honglei Ren, Yang Wang, Runnan Zhou

**Affiliations:** 1 College of Advanced Agricultural and Ecological Environment, Heilongjiang University, Harbin 150080, China; hlj_quxue1082@126.com; 2 Soybean Research Institute of Heilongjiang Academy of Agriculture Sciences, Harbin 150086, China; sobhifaid@alexu.edu.eg (S.F.L.); renguangqing2024@163.com (G.R.); syx17804588278@126.com (Y.S.); renhonglei2022@163.com (H.R.); 3 Plant Production Department, Faculty of Agriculture, Saba Basha, Alexandria University, Alexandria 21531, Egypt

**Keywords:** *Glycine max*, *Fusarium oxysporum A3*, germplasm screening, resistance genes, Northeast China, RT-qPCR

## Abstract

Soybean root rot, caused by diverse soil-borne pathogens, is a major constraint on production worldwide, with yield losses ranging from 10 to 60% under epidemic conditions. Symptomatic plants were collected from three locations in Harbin, Heilongjiang Province, China, and 23 fungal isolates were recovered using standard tissue isolation procedures. Integrated morphological characterization and rDNA-ITS sequencing identified these isolates as three *Fusarium* species: *F. oxysporum* (18 isolates, 78%), *F. equiseti* (3 isolates, 13%), and *F. brachygibbosum* (2 isolates, 9%). Pathogenicity assays following Koch’s postulates confirmed *F. oxysporum* as the predominant and most aggressive pathogen in this region. To identify resistance resources, 200 soybean germplasm accessions adapted to Northeast China were screened using an etiolated seedling hypocotyl inoculation method with *Fusarium oxysporum* isolate A3 (DSI = 68.5) as the test pathogen. Disease severity indices exhibited a continuous distribution (mean = 52.84, range = 0–100), suggesting quantitative inheritance. Accessions were classified as highly resistant (13, 6.5%), resistant (40, 20%), moderately susceptible (67, 33.5%), susceptible (43, 21.5%), or highly susceptible (37, 18.5%). To explore potential molecular mechanisms underlying resistance, RT-qPCR analysis was performed on two extreme genotypes—a highly resistant line (H9477F5, DSI = 15.3) and a highly susceptible line (HN91, DSI = 88.7) at 1, 3, and 5 days post-inoculation. The resistant line maintained consistently higher expression of positive regulators *GmFER* and *GmSOD1*, with *GmFER* reaching 15.89-fold induction at day 3. Conversely, expression of negative regulators *GmJAZ1* and *GmTAP1* remained lower in the resistant line, with susceptible plants showing 5.62-fold higher *GmJAZ1* expression at day 3. These findings provide characterized pathogen isolates, resistant germplasm resources (53 accessions with HR or R classifications), and preliminary molecular insights that may inform breeding strategies for improving root rot resistance in Northeast China.

## 1. Introduction

Soybean (*Glycine max* L.) is one of the most economically important crops globally, serving as a primary source of plant-based protein and oil for human consumption and animal feed [1]. Heilongjiang Province in Northeast China is a critical production region within one of the world’s three major black-soil belts, yet sustainable production faces increasing pressure from root-rot diseases [2]. Yield losses typically range from 10 to 60%, with complete crop failure occurring during epidemic years [3,4]. The disease is caused by a complex of soil-borne fungal pathogens, including *Phytophthora sojae*, *Rhizoctonia solani*, *Pythium* spp., and *Fusarium* spp., though pathogen composition varies considerably across regions [5,6,7].

The etiology of soybean root rot exhibits considerable regional variation due to differences in climate, soil conditions, and cultivar preferences. In Altay Prefecture, Xinjiang, *Fusarium oxysporum* was identified as the most frequently isolated and highly pathogenic species causing root rot [8]. while studies in Anhui Province documented the strong pathogenicity of *F. culmorum*, *F. proliferatum*, and *F. oxysporum* [9]. Surveys in Jilin Province recovered *F. solani*, *F. oxysporum*, *F. verticillioides*, and *F. proliferatum* from diseased roots [10], and provincial-level statistics from Heilongjiang identified *F. oxysporum* and *F. solani* as dominant species [11]. Globally, *F. oxysporum* has been recognized as a major soybean pathogen since its initial identification in Iowa in 1953 [12], with subsequent reports from Poland [13]. Brazil and other production regions confirm their widespread distribution. The pathogen induces diverse symptoms, such as seedling damping-off, root necrosis, vascular discoloration, and lodging, with documented yield reductions reaching 64%, and seed infection reduces field germination by up to 40% [14].

Interpreting *Fusarium* pathology requires caution, as the frequency of isolation does not necessarily correlate with pathogenic importance. Substantial variation in aggressiveness exists within the genus [15,16], particularly within the *F. oxysporum* species complex, where some isolates cause severe disease while others exhibit minimal pathogenic activity [17]. Rigorous pathogenicity testing following Koch’s postulates, therefore, remains essential for establishing causal relationships between isolated fungi and field disease [18]. Moreover, accurate species-level identification presents technical challenges. While the internal transcribed spacer (ITS) region serves as a standard fungal barcode, it shows recognized limitations for definitive species delimitation within certain *Fusarium* complexes, particularly the *F. incarnatum-equiseti* and *F. oxysporum* groups, where multilocus sequence typing using genes such as *TEF1-α* and *RPB2* provides more robust taxonomic resolution [19].

Developing disease-resistant cultivars represents the most economically viable and environmentally sustainable approach for managing soybean root rot [20,21]. Recent germplasm evaluations have demonstrated exploitable genetic variation for *Fusarium* resistance: screening of 105 wild soybean accessions identified 67% with resistance, and eight showing high resistance [22]; evaluation of 200 recombinant inbred lines revealed 25 with high resistance to *F. oxysporum* [23]; and screening of 153 materials identified four highly resistant varieties [24]. Despite these advances, the molecular architecture underlying resistance to *F. oxysporum* remains incompletely characterized. Several genes have been implicated in resistance responses, including those encoding antioxidant enzymes (*GmSOD1*) [25], iron homeostasis regulators (*GmFER*) [26,27], jasmonate signaling repressors (*GmJAZ1*) [28], and chromatin-modifying enzymes (*GmTAP1*) [29,30]. Expression profiling of such genes in contrasting genotypes can illuminate defense mechanisms and potentially inform marker-assisted selection.

Despite extensive *Fusarium* documentation across China, critical knowledge gaps persist for the Harbin region of Heilongjiang Province, a core soybean production area. While provincial-level surveys have identified dominant species [11], systematic morphological–molecular characterization combined with rigorous pathogenicity testing has not been conducted specifically for Harbin. Furthermore, *F. brachygibbosum*, an emerging pathogen recently documented on soybean in limited areas, has not been reported in Northeast China, leaving its pathogenic potential in this region unknown [31]. Large-scale germplasm screening data linking phenotypic resistance to molecular defense mechanisms are similarly lacking for this region. Existing resistant germplasm from other areas may lack adaptation to local conditions, particularly the black soil characteristics, cool-temperate climate, and short growing season typical of Northeast China, necessitating the identification of locally adapted resistance sources. Understanding molecular mechanisms of resistance is essential for developing effective markers to support regional breeding programs. This study addresses these gaps through three integrated objectives: first, to identify and characterize *Fusarium* species associated with soybean root rot in Harbin through combined morphological analysis, ITS sequencing, phylogenetic analysis, and pathogenicity validation via Koch’s postulates; second, to screen 200 locally adapted soybean germplasm accessions for resistance to the primary pathogen; and third, to explore molecular resistance mechanisms through gene expression analysis in contrasting genotypes. These findings provide practical resources characterized pathogen isolates and resistant germplasm to support breeding efforts for enhanced root rot resistance in Northeast China.

## 2. Results

### 2.1. Isolation and Molecular Identification of Fusarium Species from Diseased Soybean

**Figure 1:**
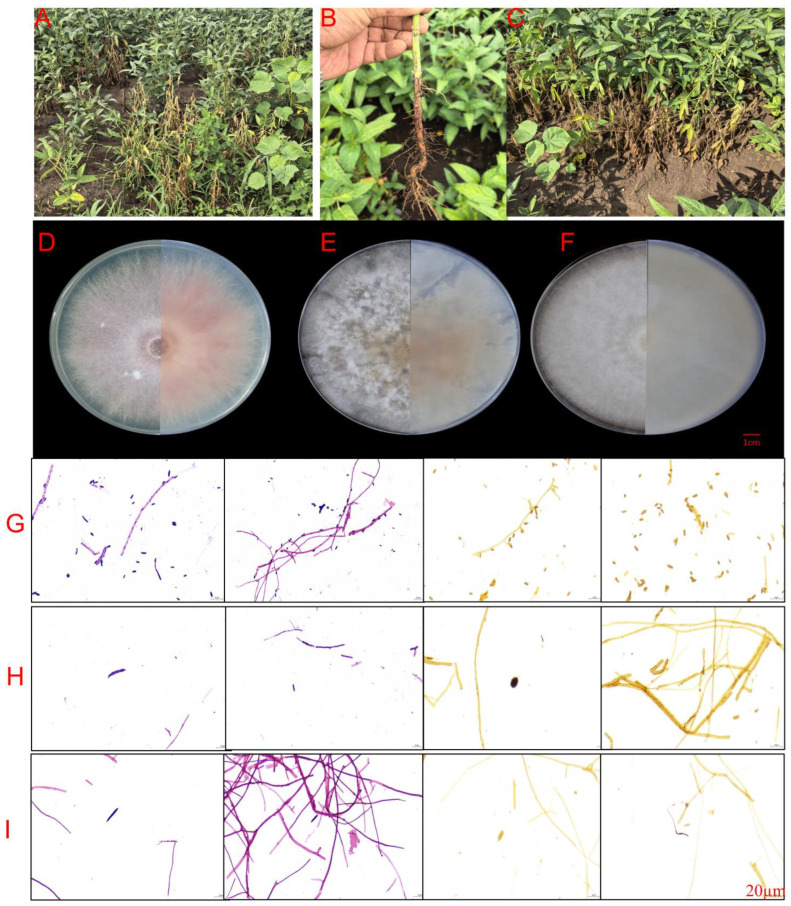
Field symptoms and colony morphology of *Fusarium* species isolated from soybean root rot in Harbin, Northeast China. (**A**–**C**) Field symptoms at three collection sites showing characteristic root rot syndrome: stunted plants with wilting foliage, root necrosis, and reduced stand density. (**D**–**F**) Colony morphology on potato dextrose agar after 7 days at 26 °C, showing both obverse (upper surface) and reverse (underside) views. (**D**) *Fusarium oxysporum* isolate A3 (Type I) displaying characteristic white to pinkish-purple coloration with flocculent aerial mycelium (obverse) and pale pink to purplish-red pigmentation (reverse); (**E**) *F. equiseti* isolate B2 (Type II) showing yellowish-brown pigmentation with cotton-like texture and orange center; (**F**) *F. brachygibbosum* (Type III isolate) exhibiting light brown, velvety colony texture. Scale bar in panel F = 1 cm (applies to **D**–**F**). (**G**–**I**) Microscopic features visualized through light microscopy. (**G**) *F. oxysporum* isolate A3: septate hyphae (**left panels**) and characteristic microconidia and macroconidia (**right panels**) showing oval to kidney-shaped microconidia and fusiform, slightly curved macroconidia with 3–5 septa; (**H**) *F. equiseti*: septate hyphae with occasional annular constrictions (left panels) and ellipsoidal microconidia with verrucose chlamydospores (right panels); (**I**) *F. brachygibbosum*: organized mycelial bundles (left panels) and notably curved macroconidia (right panels) with deep pigmentation. Scale bar in panel I = 20 μm (applies to **G**–**I**).

In June 2025, symptomatic soybean plants were collected from three locations in Harbin City, Heilongjiang Province: Minzhu Town (45°45′ N, 126°38′ E), Changling Lake (45°52′ N, 126°42′ E), and Xiangyang Farm (45°38′ N, 126°35′ E). Diseased plants displayed characteristic root rot symptoms, including red-brown depressed necrotic lesions on roots and lower stems, lesion expansion encircling primary roots, epidermal decay, sparse lateral root development, and, in severe cases, complete root blackening with wet rot. Above-ground symptoms included stunting, wilting, premature leaf abscission, chlorosis of cotyledons and lower leaves, and reduced branching (Figure 1A–C). From 30 diseased plants (10 per location), 23 fungal isolates were obtained through tissue isolation and single-spore purification. Based on colony morphology on potato dextrose agar (PDA), isolates segregated into three distinct types: Type I (*n* = 18, 78%) produced white to pinkish-purple flocculent colonies; Type II (*n* = 3, 13%) formed yellowish-brown colonies with cotton-like texture and orange centers; and Type III (*n* = 2, 9%) developed light brown velvety colonies (Figure 1D–I and Table S1). To verify morphological groupings, genomic DNA were extracted from eight representative isolates spanning all three types and all collection locations: five Type I isolates (A1, A3 from Minzhu Town; B4, B7 from Xiangyang Farm; C6 from Changling Lake), two Type II isolates (B2 from Xiangyang Farm; C3 from Changling Lake), and one Type III isolate (A6 from Minzhu Town).

**Figure 2:**
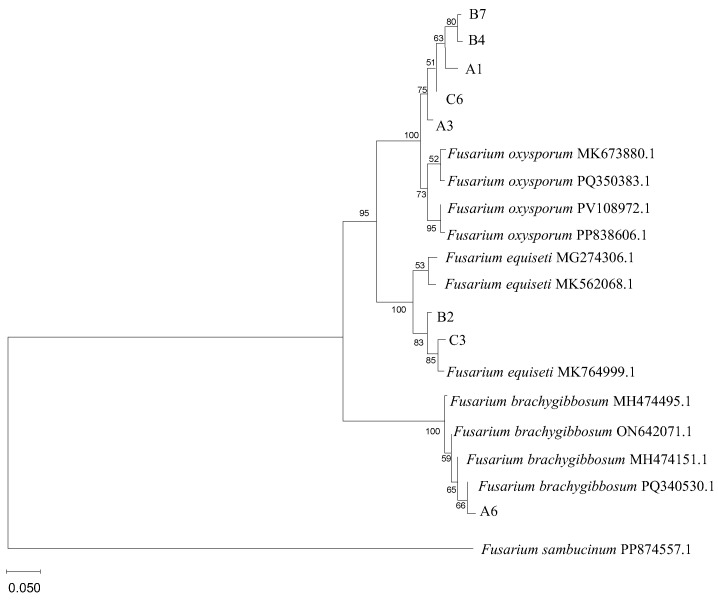
Phylogenetic tree of *Fusarium* isolates based on ITS sequences. Neighbor-Joining phylogenetic tree constructed using ITS rDNA sequences from eight representative isolates and reference strains from GenBank. *Fusarium sambucinum* (PP874557.1) was used as the outgroup. Bootstrap values (1000 replicates) are shown at branch nodes; values ≥ 70% indicate strong support. Scale bar represents 0.050 nucleotide substitutions per site. Three distinct clades correspond to *F. oxysporum* (isolates A1, A3, B4, B7, C6), *F. equiseti* (isolates B2, C3), and *F. brachygibbosum* (isolate A6). Based on both morphological and molecular data, the 23 isolates were identified as follows: 18 isolates as *Fusarium oxysporum* (78%), 3 as *Fusarium equiseti* (13%), and 2 as *Fusarium brachygibbosum* (9%) (Table 1). The high prevalence of *F. oxysporum* indicates it is the dominant pathogen causing soybean root rot in the Harbin region, consistent with previous reports from other areas of Northeast China.

Amplification and bidirectional sequencing of the internal transcribed spacer (ITS) region yielded ~550 bp products (Figure S1). BLASTn NCBI analysis against GenBank showed all sequences shared ≥99% identity with authenticated *Fusarium* species references, with query coverage ≥ 94% (Figures S2–S4). Type I isolates matched *Fusarium oxysporum* references (99–100% identity; GenBank accessions MK673880.1, PQ350383.1, PV108972.1, PP838606.1). Type II isolates matched *Fusarium equiseti* (99% identity; MK764999.1, MK562068.1, MG274306.1). The Type III isolate aligned with *Fusarium brachygibbosum* (99% identity; PQ344053.1). Neighbor-Joining phylogenetic analysis with 1000 bootstrap replicates revealed three strongly supported monophyletic clades (bootstrap ≥ 99%) corresponding to morphological types (Figure 2). Maximum Likelihood analysis yielded a congruent topology with similar support values. The remaining 15 isolates were assigned to species based on morphological concordance with sequenced representatives: 13 additional isolates matched Type I morphology (*F. oxysporum*), one matched Type II (*F. equiseti*), and one matched Type III (*F. brachygibbosum*). All ITS sequences were deposited in GenBank (accession numbers in Table S2). The final species composition comprised *F. oxysporum* (18 isolates, 78%; recovered from all three locations), *F. equiseti* (3 isolates, 13%; from Xiangyang Farm and Changling Lake), and *F. brachygibbosum* (2 isolates, 9%; from Minzhu Town only) (Table 1). The dominance of *F. oxysporum* isolate A3 across all sampling locations, combined with its subsequent identification as the most aggressive pathogen, indicates this species drives the disease complex in the Harbin region. Notably, this represents the first documentation of *F. brachygibbosum* on soybean in Northeast China, extending the known geographic range of this emerging pathogen.

MT = Minzhu Town; XF = Xiangyang Farm; CL = Changling Lake. Disease severity index from pathogenicity assays on susceptible cultivar Dongnong 50 at 14 dpi. Classification based on DSI: highly pathogenic (≥60), moderately pathogenic (50–60), weakly pathogenic (<50). Note: The 23 isolates represent three *Fusarium* species; isolation frequencies indicate relative abundance in the sampled pathogen population. Species assignments confirmed by ITS sequencing and phylogenetic analysis (Figure 2).

### 2.2. Pathogenicity Assessment and Fulfillment of Koch’s Postulates

**Figure 3:**
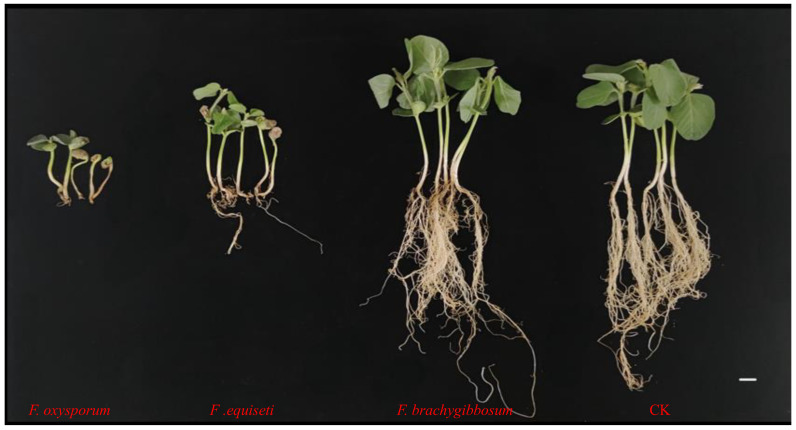
Comparative pathogenicity of three *Fusarium* species on susceptible soybean cultivar Dongnong 50. Disease symptoms at 14 days post-inoculation, demonstrating differential aggressiveness among species. Seedlings were inoculated using sorghum grain inoculum (30 g colonized grains mixed with 270 g sterile vermiculite per pot) and grown under controlled conditions (25 ± 2 °C, 16 h photoperiod, 60–70% RH). Left to right: (**1**) *F. oxysporum* (isolate A3): Severe root rot with extensive tissue necrosis and blackening, sparse or absent lateral roots, severely stunted growth, wilted chlorotic foliage, and frequent plant death (DSI = 68.5 ± 3.2, highly pathogenic). (**2**) *F. equiseti* (isolate B2): Moderate disease symptoms with partial root necrosis, reduced lateral root density, moderate stunting, and chlorotic wilted leaves (DSI = 52.3 ± 2.8, moderately pathogenic). (**3**) *F. brachygibbosum* (isolate A6): Mild symptoms with slight browning of some fibrous roots, predominantly green foliage, minimal stunting, and viable plants (DSI = 38.7 ± 2.1, weakly pathogenic). (**4**) Non-inoculated control (CK): Healthy seedlings with vigorous growth, dark green foliage, well-developed root systems with abundant lateral roots, and no disease symptoms (DSI = 1.0 ± 0.6). Scale bar = 2 cm.

To establish causal relationships between isolated fungi and disease symptoms, representative isolates from each species (*F. oxysporum* isolate A3, *F. equiseti* B2, *F. brachygibbosum* A6) were tested for pathogenicity on the susceptible cultivar Dongnong 50, using sorghum grain inoculum. Disease severity was evaluated at 14 days post-inoculation (dpi) using a 0–7 scale, and morphometric measurements quantified growth impacts. All three species caused root-rot symptoms, but pathogenicity varied significantly (*p* < 0.001; Figure 3). *Fusarium oxysporum* exhibited the highest aggressiveness, with a disease severity index (DSI) of 68.5 ± 3.2, indicating it is highly pathogenic (DSI ≥ 60). Infected seedlings displayed severe symptoms, including extensive root necrosis and breakage, sparse lateral roots, stunted growth, severe chlorosis, wilted leaves, and frequent seedling death. In extreme cases, cotyledons failed to unfold, hypocotyl tissues blackened, and seedlings died before establishing normal growth. Morphometric analysis confirmed severe growth suppression: compared to controls, *F. oxysporum* isolate A3-infected plants showed a 45.3% reduction in height, a 52.1% reduction in shoot weight, a 61.8% reduction in root weight, 48.7% shorter primary roots, and 68.2% fewer lateral roots (Table S3). The pathogen was successfully re-isolated from symptomatic tissues and confirmed morphologically and molecularly identical to the original isolate (ITS sequence ≥ 99% identity), fulfilling Koch’s postulates.

*Fusarium equiseti* demonstrated moderate pathogenicity (DSI = 52.3 ± 2.8; 50 ≤ DSI < 60). While less severe than *F. oxysporum* isolate A3, substantial damage occurred: plant height decreased 30.4%, shoot weight declined 38.9%, root weight dropped 45.3%, with corresponding reductions in root length and lateral root density. Seedlings exhibited chlorosis and wilting but remained viable, showing impaired growth rather than mortality. Root systems displayed moderate necrosis with reduced main and lateral root development. *Fusarium brachygibbosum* exhibited weak pathogenicity (DSI = 38.7 ± 2.1; DSI < 50). Plant growth appeared relatively normal, with minimal visible differences compared to controls. Above-ground tissues remained predominantly green with well-expanded leaves. Root systems appeared largely healthy, though some fibrous roots showed slight browning. Growth reductions were modest: 15.2% decrease in height with corresponding minor reductions in other parameters. This species appears to function as a minor or opportunistic pathogen under tested conditions. Non-inoculated control plants showed normal growth with healthy green foliage, well-developed root systems, and no disease symptoms (DSI = 1.0 ± 0.6). The pathogenicity ranking (*F. oxysporum* isolate A3 > *F. equiseti* > *F. brachygibbosum*; all pairwise comparisons *p* < 0.01 by Tukey’s HSD) corresponds to isolation frequency (78% > 13% > 9%), suggesting that the most aggressive pathogen dominates the disease complex. This correlation, while not definitive proof of field prevalence, indicates *F. oxysporum* isolate A3 likely drives disease development in Harbin soybean production systems.

### 2.3. Screening of Soybean Germplasm for Resistance to F. oxysporum Isolate A3

**Figure 4:**
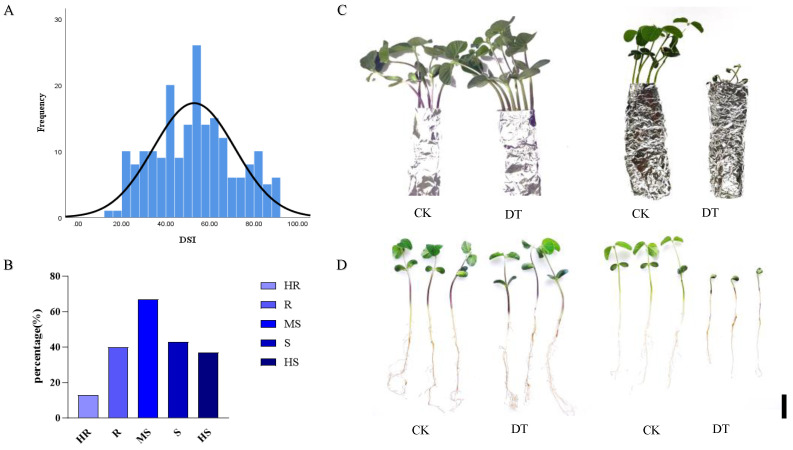
Germplasm screening of 200 soybean accessions for resistance to *Fusarium oxysporum* isolate A3. (**A**) Frequency distribution of disease severity indices (DSIs) showing quantitative variation in resistance. (**B**) Distribution of accessions across five resistance categories. (**C**) Representative phenotypes of resistant and susceptible accessions at 5 days post-inoculation. Scale bar = 2 cm. (**D**) Root and hypocotyl symptoms demonstrating differential resistance responses. Scale bar = 2 cm. Experimental details: Each accession was evaluated with 30 seedlings in total (15 per experiment × 2 independent experiments = 5 per pot × 3 replicate pots × 2 experiments). Two check cultivars were included in each of 10 screening batches: Dongnong 50 (susceptible standard, DSI = 61.2 ± 3.4) and ‘Tiedou 44’ (resistant standard, DSI = 22.3 ± 2.1). The continuous distribution of resistance phenotypes suggests that breeding strategies should focus on pyramiding multiple moderate-effect resistance alleles rather than single major genes. Complete resistance data for all 200 accessions are presented in Table S4.

Given that *F. oxysporum* isolate A3s has been identified as the dominant and most aggressive pathogen, a large-scale germplasm screening was conducted to identify resistance sources for breeding programs. A 200 soybean accessions (165 released cultivars and 35 advanced breeding lines) adapted to Northeast China were evaluated using an etiolated-seedling hypocotyl inoculation method with the *F. oxysporum* isolate A3 as the challenge pathogen. Disease severity indices ranged from 2.3 to 97.8 (mean = 52.84 ± 1.2; Figure 4A). The DSI distribution was continuous and approximately normal (Shapiro–Wilk test W = 0.987, *p* = 0.18), indicating quantitative inheritance likely involving multiple genes. No immune accessions (DSI = 0) were identified; the most resistant accession (H9477F5) showed DSI = 15.3, suggesting complete resistance is rare or absent in the evaluated germplasm. Based on DSI values, accessions were classified into five categories: highly resistant (HR, DSI ≤ 20; 13 accessions, 6.5%), resistant (R, 20 < DSI ≤ 40; 40 accessions, 20%), moderately susceptible (MS, 40 < DSI ≤ 60; 67 accessions, 33.5%), susceptible (S, 60 < DSI ≤ 80; 43 accessions, 21.5%), and highly susceptible (HS, DSI >80; 37 accessions, 18.5%) (Figure 4B; complete data in Tables S4–S6). The screening revealed substantial genetic variation for resistance. Resistant accessions (HR + R) collectively comprised 26.5% of evaluated materials, providing valuable breeding resources. However, 40% of accessions were susceptible or highly susceptible (S + HS), with an additional 33.5% showing intermediate responses (MS), indicating overall vulnerability of current germplasm to *F. oxysporum* isolate A3 root rot. Several resistant accessions showed consistent performance across replicates, including ‘Tiedou 44’ (DSI = 22.3, resistant check), ‘Jiadou 25’ (DSI = 19.4), and ‘Suinong 20’ (DSI = 23.1), all of which have been previously reported to possess multiple disease resistances.

Phenotypic comparison of extreme genotypes clearly demonstrated differences in resistance (Figure 4C,D). Highly resistant accessions exhibited vigorous growth with dark green, fully expanded leaves, similar to those of non-inoculated controls. Inoculated hypocotyls displayed only slight superficial browning at inoculation sites without tissue necrosis or lesion expansion, and root systems remained healthy and well-developed. Conversely, highly susceptible accessions showed severe symptoms, including wilted unexpanded cotyledons, extensive hypocotyl necrosis with lesions extending upward and downward from inoculation sites, complete hypocotyl rot, and severely stunted or absent root development. In extreme cases, seedlings died within 5 days post-inoculation.

The continuous distribution of resistance phenotypes, the absence of discrete resistance classes, and the normal distribution pattern indicate that resistance to the *F. oxysporum* isolate A3 follows a quantitative genetic model. This suggests that breeding strategies should focus on pyramiding multiple moderate-effect resistance alleles from diverse genetic sources rather than relying on a single major resistance gene. The 53 identified resistant accessions (HR + R categories) provide genetic resources for QTL mapping, marker-assisted selection, and cultivar development programs to enhance root rot resistance in Northeast China.

### 2.4. Gene Expression Profiling in Contrasting Genotypes

**Figure 5:**
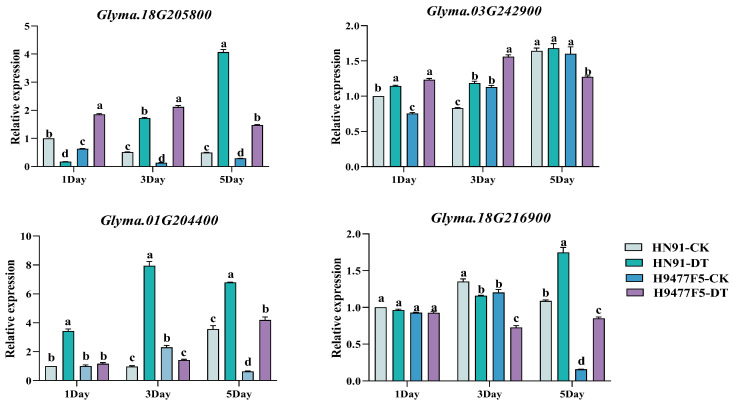
Expression profiles of defense-related genes in resistant and susceptible soybean genotypes following *F. oxysporum* isolate A3 inoculation. Relative expression levels of four candidate genes were determined by quantitative RT-PCR at 1, 3, and 5 days post-inoculation (dpi) in contrasting genotypes. Two extreme genotypes were selected from germplasm screening: H9477F5-CK = non-inoculated resistant control, H9477F5-DT = *F. oxysporum* isolate A3-inoculated resistant line, HN91-CK = non-inoculated susceptible control, HN91-DT = *F. oxysporum* isolate A3-inoculated susceptible line. Expression was normalized to reference gene *GmActin11* and calculated relative to genotype-matched non-inoculated controls at 0 dpi (pre-inoculation baseline) using the 2^−ΔΔCt^ method. Different lowercase letters above bars indicate significant differences between genotypes within each time point.

To explore molecular mechanisms underlying differential resistance, two extreme genotypes from the germplasm screen were selected for gene expression analysis: highly resistant H9477F5 (DSI = 15.3 ± 1.8) and highly susceptible HN91 (DSI = 88.7 ± 3.2). Four defense-related genes were analyzed at 1, 3, and 5 days post-inoculation (dpi), each representing different defense mechanisms: *GmFER* (*Glyma.18G205800*), which is involved in iron homeostasis and managing oxidative stress; *GmSOD1* (*Glyma.03G242900*), a superoxide dismutase that detoxifies reactive oxygen species; *GmJAZ1* (*Glyma.01G204400*), a repressor in jasmonate signaling; and *GmTAP1* (*Glyma.18G216900*), a histone acetyltransferase that acts as a susceptibility factor. Expression levels were quantified by RT-qPCR with *GmActin11* as the reference gene, comparing inoculated plants to genotype-matched non-inoculated controls. Positive regulators showed elevated expression in resistant genotypes. Both *GmFER* and *GmSOD1* showed consistently higher expression in H9477F5 than in HN91 across all time points (Figure 5). *GmFER* expression showed dramatic genotype-specific kinetics: in H9477F5, expression peaked rapidly at 3 dpi (15.89-fold induction relative to controls), then declined to near-baseline by 5 dpi (4.2-fold), suggesting successful resolution of oxidative challenge. In contrast, HN91 showed delayed peak induction at 5 dpi (8.25-fold) with sustained elevation, possibly reflecting ongoing tissue damage and failed containment. The earlier and stronger *GmFER* response in resistant plants indicates rapid activation of iron sequestration mechanisms to limit infection-induced oxidative stress by restricting Fenton chemistry. *GmSOD1* displayed moderate but sustained upregulation in both genotypes, with H9477F5 maintaining consistently higher levels throughout the time course. At the critical 3 dpi time point, *GmSOD1* expression in H9477F5 was 1.32-fold higher than in HN91. The resistant line showed relatively stable expression across time points (1.1–1.6-fold induction), suggesting constitutive readiness of antioxidant defenses. The coordinated upregulation of both *GmFER* and *GmSOD1* in resistant plants indicates that efficient management of reactive oxygen species (ROS) constitutes a key resistance mechanism. Negative regulators showed suppressed expression in resistant genotypes. In contrast to positive regulators, *GmJAZ1* and *GmTAP1* exhibited consistently lower expression in H9477F5 than in HN91 (Figure 5). *GmJAZ1*, encoding a jasmonic acid (JA) signaling repressor, showed dramatically elevated expression in the susceptible line, particularly at 3 dpi, where HN91 reached 8.0-fold induction—5.62 times higher than H9477F5 (1.42-fold). High *GmJAZ1* expression in susceptible plants likely suppresses JA-mediated defenses, potentially facilitating pathogen colonization through active pathogen manipulation or by preventing host cells from degrading JAZ repressor proteins. By 5 dpi, *GmJAZ1* expression in HN91 declined to 6.8-fold but remained substantially elevated compared to H9477F5 (4.1-fold).

*GmTAP1* expression patterns similarly distinguish resistant from susceptible genotypes. At 1 dpi, both genotypes showed baseline expression (~1.0-fold). However, by 3 dpi, *GmTAP1* expression in HN91 increased to 1.35-fold, whereas it remained at baseline in H9477F5. The difference became most pronounced at 5 dpi, with HN91 showing 1.7-fold induction—2.05 times higher than H9477F5 (0.83-fold, slightly below baseline). *GmTAP1* encodes a histone acetyltransferase previously identified as a susceptibility factor in the soybean-*Phytophthora sojae* pathosystem, where pathogen effectors recruit it to activate susceptibility genes through chromatin remodeling. Lower *GmTAP1* expression in resistant plants may restrict pathogen manipulation of host gene expression. The 3 dpi time point represents a critical decision point in infection outcomes. At this time point, expression differences between resistant and susceptible genotypes were most pronounced for three of four genes examined (*GmFER*, *GmJAZ1*, *GmTAP1*). The resistant line exhibited strong upregulation of defense genes (*GmFER*, *GmSOD1*) while maintaining low expression of susceptibility factors (*GmJAZ1*, *GmTAP1*), suggesting coordinated defense activation. Conversely, the susceptible line showed delayed and weaker induction of defense genes accompanied by strong expression of negative regulators, potentially creating a permissive environment for pathogen colonization.

These expression patterns suggest resistance to *F. oxysporum* isolate A3 involves multiple integrated defense layers: enhanced oxidative stress management through coordinated action of ferritin (iron sequestration) and superoxide dismutase (ROS detoxification); maintenance of JA signaling competence through suppression of *GmJAZ1* repressor; and restriction of pathogen-mediated chromatin manipulation through low *GmTAP1* expression. The temporal coordination of these responses, particularly rapid early *GmFER* induction and constitutive *GmSOD1* readiness, appears critical for successful defense. However, as this analysis examined only two genotypes representing phenotypic extremes, validation across additional resistant and susceptible accessions is necessary to confirm these patterns as consistent molecular signatures of resistance.

## 3. Discussion

### 3.1. Fusarium Species Diversity and Pathogenic Potential in Harbin

The molecular documentation of *Fusarium* species composition in Harbin fills a regional knowledge gap while revealing both expected and novel findings. The dominance of *F. oxysporum* isolate A3 (78% of isolates) aligns with broader surveys in Heilongjiang [12] and global reports that position this species as a primary soybean root pathogen from Iowa to Brazil [13,14,15]. However, the co-occurrence of *F. equiseti* (13%) and *F. brachygibbosum* (9%) merits discussion, particularly given their contrasting pathogenic profiles. *F. brachygibbosum* detection represents the first report of this species on soybean in Northeast China. Previously documented in limited geographic areas, its presence in Harbin raises questions about whether it represents range expansion or historical oversight. The weak pathogenicity (DSI 38.7) might explain previous non-detection if surveys relied solely on isolation from severely diseased plants; less aggressive pathogens would be outcompeted during tissue colonization. Alternatively, *F. brachygibbosum* may function opportunistically, colonizing tissues pre-damaged by more aggressive species. The ecological role of this species in soybean cropping systems requires clarification. *F. equiseti* presents intriguing complexity. The pathogenicity assays demonstrated clear disease induction (DSI 52.3, 30% growth reduction), establishing isolate B2 as a bona fide pathogen under controlled conditions. Yet recent literature documents *F. equiseti* strains functioning as plant growth-promoting fungi. Feng et al. [32] showed that inoculation with *F. equiseti* altered ryegrass rhizosphere communities, enhancing salt tolerance and growth. Others report biocontrol activity against diverse pathogens and endophytic colonization, conferring drought resistance. This apparent contradiction, pathogen versus mutualist, likely reflects substantial intraspecific variation. Several factors may determine whether a given *F. equiseti* strain acts pathogenically or beneficially: strain-specific genetic differences in secreted metabolite and effector repertoires; host plant species and genotype compatibility; plant developmental stage and physiological state; environmental stressors modulating plant-microbe interactions; microbial community context, with competitive or facilitative interactions influencing outcomes. The isolate B2, inoculated at high density onto wounded, susceptible seedlings under sterile conditions, exhibited clear pathogenicity. Whether the same strain would benefit mature plants, different genotypes, or plants under field conditions with complex microbial communities remains unknown.

This ecological duality carries management implications. Indiscriminate targeting of all *F. equiseti* may be counterproductive if beneficial strains provide biocontrol or stress-tolerance services. Future research should prioritize developing molecular markers to distinguish pathogenic from beneficial *F. equiseti* strains; elucidating environmental and host factors that determine colonization outcomes; and, potentially, harnessing beneficial strains for biological control while managing pathogenic ones. The balance between pathogenic risk and mutualistic benefit may prove context-dependent, requiring nuanced rather than blanket approaches. Comparing these findings with global surveys reveals both conserved and region-specific patterns. *F. oxysporum* isolate A3 dominance in soybean root rot occurs worldwide United States [12], Poland [13], Brazil, and multiple Chinese provinces [9,33], suggesting fundamental compatibility between this species and soybean. However, co-occurring species vary substantially by region: *F. solani* and *F. proliferatum* dominate in southern China [10,12]; *F. graminearum* is found in temperate regions of North America; F. verticillioides predominates in warmer subtropical regions. These assemblage differences likely reflect climate (temperature and precipitation regimes), edaphic factors (soil pH, texture, organic matter), cropping system history (rotation patterns, tillage practices), and historical pathways of pathogen introduction.

An additional complexity concerns *F. oxysporum* formae speciales—host-specialized lineages within the species complex. While it demonstrated strong pathogenicity (DSI 68.5), *F. oxysporum* comprises numerous formae speciales with distinct host ranges. Whether soybean-associated *F. oxysporum* in Harbin represents a specialized forma specialis or comprises generalist strains capable of infecting multiple hosts remains unresolved. The reliance on ITS sequences, while adequate for species-level identification (≥99% identity to authenticated references), cannot resolve subspecific structure. Multilocus sequencing using TEF1-α and RPB2 would clarify phylogenetic placement within the *F. oxysporum* species complex and potentially identify markers for rapid strain identification. The pathogenic ranking (*F. oxysporum* isolate A3 > *F. equiseti* > *F. brachygibbosum*) mirrors isolation frequency, suggesting that more aggressive pathogens dominate diseased tissue. However, isolation frequency may not reflect field prevalence if competitive interactions during tissue colonization favor faster-growing species. Molecular surveys using species-specific qPCR primers could quantify relative abundance in field soils independent of cultivation biases, clarifying whether *F. oxysporum* isolate A3 dominance represents true ecological prevalence or an isolation artifact. Globally, *Fusarium* root rot imposes substantial economic burdens. In the United States, *Fusarium* species cause estimated annual losses of 10–15 million bushels, with epidemic years exceeding 30 million in major production states. Brazil, the world’s largest soybean producer, ranks Fusarium root rot among the top five disease constraints, particularly in the Cerrado, where intensive monoculture and warm soils favor pathogen proliferation. In China, where soybean production has declined in part due to disease pressures, *Fusarium* root rot represents a significant barrier to achieving soybean self-sufficiency goals outlined in national agricultural policy. The disease proves particularly problematic under continuous soybean cropping, where pathogen inoculum accumulates. *F. oxysporum* isolate A3 persists as chlamydospores in soil for years, making rotational control challenging. In typical 3–4-year rotations in Northeast China, *F. oxysporum* isolate A3 populations can survive rotation cycles, with rapid population expansion following soybean reintroduction. The present documentation of *F. oxysporum* dominance in Harbin aligns with this pattern and suggests that current cropping systems may inadvertently select for this species’ persistence. Management strategies must account for long-term inoculum dynamics rather than focusing solely on single-season interventions.

### 3.2. Genetic Variation for Resistance and Breeding Implications

The identification of 53 resistant accessions (26.5% of screened materials) demonstrates exploitable genetic variation within adapted Northeast China germplasm. This frequency compares favorably with other screening efforts: 67% resistance among wild soybean accessions [34], 12.5% among recombinant inbred lines, and 2.6% among diverse collections [35,36]. The higher resistance frequency in this panel likely reflects enrichment for elite cultivars that have already undergone indirect selection for stress tolerance and agronomic performance under Northeast China production conditions. Several accessions merit particular emphasis. Tiedou 44, which confirmed resistance in these assays, carries documented resistance to soybean mosaic virus, demonstrating broad-spectrum disease resistance potentially mediated by shared immune components [37]. Jiadou 25 exhibits resistance to frogeye leaf spot (*Cercospora sojina*) and soybean cyst nematode (*Heterodera glycines*), alongside drought tolerance [38], a combination suggesting robust stress response networks. Suinong 20’s resistance to multiple foliar and root pathogens hints at systemic defense competence. Whether these multi-pathogen resistances reflect linked loci, pleiotropic gene action, or pyramided resistance alleles requires genetic dissection, but the phenomenon suggests these varieties as particularly valuable breeding parents.

The continuous DSI distribution (mean 52.84, range 5.2–100) and the approximate normality indicate polygenic control consistent with quantitative trait locus mapping studies that identify multiple moderate-effect resistance loci for *Fusarium* diseases [35,36]. The absence of immune accessions (minimum DSI 5.2) suggests complete resistance may be rare or absent in cultivated germplasm, possibly because complete resistance mechanisms impose fitness costs under pathogen-free conditions. This contrasts with race-specific resistance to *Phytophthora sojae*, where single *Rps* genes confer near-immunity. Polygenic resistance architecture carries implications for breeding. Simple backcrossing to introgress major resistance genes proves effective for race-specific resistances but may inadequately capture quantitative resistance. Instead, breeders should consider: Marker-assisted recurrent selection to increase favorable allele frequencies across multiple resistance loci. Genomic selection using genome-wide markers to predict breeding values for complex resistance. Allele pyramiding strategies combining moderate-effect loci from diverse sources, potentially the 53 resistant accessions identified here, to achieve transgressive segregation exceeding parental resistance levels. (4) Maintaining genetic diversity rather than fixing elite allele combinations, as genetic heterogeneity may buffer against pathogen adaptation.

The susceptible fraction (55% as MS, S, or HS) highlights germplasm vulnerability, emphasizing the breeding program’s urgency. Many cultivars likely lack sufficient resistance alleles, making them vulnerable under high disease pressure. The distribution shows continuous variation, suggesting resistance is better viewed as a quantitative trait rather than as discrete classes like HR, R, MS, S, HS, which impose artificial boundaries. Breeders should select for incremental improvements over generations. Field validation of resistant lines is crucial, as lab and greenhouse tests may not accurately predict field performance under multiple stressors. Multi-environment testing across Northeast China’s diverse zones will confirm whether resistance persists and how genotype-by-environment interactions affect it.

### 3.3. Preliminary Molecular Insights and Future Transcriptomic Directions

The gene expression analysis, though limited to two genotypes and four candidate genes, reveals intriguing patterns warranting discussion and follow-up. The differential expression of *GmFER, GmSOD1, GmJAZ1,* and *GmTAP1* between the resistant H9477F5 and the susceptible HN91 suggests that these genes participate in resistance responses, though this represents correlation rather than causation, given the absence of functional validation. *GmSOD1*, controlled by the transcription factor *GmZFP03*, is upregulated during *Phytophthora sojae* resistance, thereby enhancing antioxidant capacity [28]. *GmFER*, a ferritin family member regulating iron homeostasis, responds to phosphorus deficiency and iron toxicity [30], with overexpression enhancing Arabidopsis root growth and iron tolerance [29]. Elevated expression of both genes in resistant plants suggests that oxidative stress management is a component of resistance, a common theme in plant-pathogen interactions, where infection triggers the production of reactive oxygen species. Whether this response actively suppresses *F. oxysporum* growth or represents collateral defense activation remains unclear.

*GmJAZ1* and *GmTAP1* function as negative regulators, and their lower expression in resistant plants is consistent with the expectation that pathogen virulence induces these susceptibility factors. *GmJAZ1*, a jasmonate signaling repressor, suppresses JA-mediated stress responses by inhibiting MYC2 transcription factors [39]. *Phytophthora sojae* effector *Avh94* stabilizes *GmJAZ1*, blocking degradation and disrupting JA signaling to promote infection [40], a paradigm for effector-mediated susceptibility. Whether *F. oxysporum* employs analogous strategies remains speculative, but the high *GmJAZ1* expression in susceptible plants (8.0-fold at 3 dpi) hints at either pathogen manipulation or endogenous regulatory dysfunction.

*GmTAP1*, a histone acetyltransferase, exemplifies a characterized susceptibility factor. *P. sojae* effector *PsAvh52* recruits *GmTAP1* to activate susceptibility gene expression via chromatin acetylation [29], with CRISPR/Cas9 knockouts conferring resistance without agronomic penalties [41]. The lower *GmTAP1* expression in resistant H9477F5 (0.83-fold at 5 dpi, compared with HN91’s 1.7-fold) suggests limited activation of susceptibility genes, potentially restricting pathogen manipulation of host transcription. While these gene functions have been studied mainly in Phytophthora interactions, their differential expression during Fusarium infection indicates conserved roles across pathosystems. Plants probably use core defense mechanisms, such as managing oxidative stress, hormone signaling, and chromatin remodeling, against various pathogens, with pathogen-specific adaptations built on this shared machinery. Whether Fusarium species have effectors that target *GmJAZ1* or *GmTAP1*, as Phytophthora effectors do, remains an open and approachable question for future effector-omics research. The 3-dpi time point proved most informative, showing the greatest expression differences among the four genes. This timing aligns with known defense response stages: early recognition and signaling (0–24 h), peak transcriptional changes (24–72 h), and then a shift toward resistance or susceptibility (72–120 h). Sampling at 1 dpi detected early responses with only modest genotypic differences, indicating similar initial pathogen recognition. The divergence observed by 3 dpi suggests that resistance is determined during the defense amplification phase rather than during recognition. By 5 dpi, expression levels in H9477F5 began to return toward baseline (especially *GmFER*), suggesting successful containment of infection, whereas HN91 maintained higher expression, possibly reflecting ongoing colonization and tissue damage.

This work’s limitations include analyzing only four genes across two genotypes, which is insufficient to fully understand resistance mechanisms. A systematic approach would involve pathway analysis of entire pathways rather than individual genes, including multiple isoforms and enzymes; studying all resistance-related pathways such as jasmonate, salicylate, and ethylene; testing additional resistant and susceptible genotypes to determine if expression patterns are consistent or specific; sampling at more time points to better capture defense dynamics; using genome-wide profiling like RNA-seq to discover new resistance genes; and performing functional validation through genetic experiments to confirm the roles of candidate genes. Hypothesis-generating observations that justify comprehensive follow-up rather than definitive mechanistic conclusions. The expression patterns suggest resistance works through coordinated regulation across multiple defense layers, oxidative stress management, hormone signaling, and chromatin state, but thorough validation remains necessary. Future work should also integrate host transcriptomics with pathogen transcriptomics to quantify fungal biomass and gene expression during infection. This dual perspective would clarify whether observed changes in plant gene expression actively suppress pathogen growth or reflect ineffective defenses against ongoing colonization. Pathogen virulence gene expression profiling could identify effectors targeting *GmJAZ1, GmTAP1*, or other susceptibility factors, thereby enabling effector-guided breeding or genome-editing strategies. Developing molecular markers for resistance breeding ultimately requires linking gene expression patterns to DNA polymorphisms (QTLs or causative variants). Expression QTL mapping in segregating populations derived from resistant × susceptible crosses could identify regulatory variants controlling defense gene expression. Alternatively, genome-wide association studies using the 200 screened accessions as a population might identify resistance-associated loci, with candidate gene expression providing biological validation. Despite these limitations and future needs, these preliminary findings show that molecular validation, even on a small scale, complements phenotypic screening and provides hypotheses for mechanism-focused research. The 53 resistant accessions identified are valuable breeding resources regardless of whether the four genes analyzed prove causally influential; genetic improvement can proceed through phenotypic selection while mechanistic understanding develops concurrently.

## 4. Materials and Methods

### 4.1. Pathogen Isolation from Field Samples

A field sampling was conducted in June 2025 at three locations in Harbin City, Heilongjiang Province, China: Minzhu Town (45°45′ N, 126°38′ E), Changling Lake (45°52′ N, 126°42′ E), and Xiangyang Farm (45°38′ N, 126°35′ E). At each location, 10 symptomatic plants exhibiting characteristic root-rot symptoms (root necrosis, vascular discoloration, wilting, and chlorosis) were collected, yielding 30 plants in total. Plants were transported to the laboratory in sealed plastic bags with ice packs and processed within 24 h. Diseased roots were washed under running tap water to remove soil, then surface-sterilized by sequential immersion in 75% ethanol (30 s), 2% sodium hypochlorite (2 min), and three rinses with sterile distilled water. Tissue segments (approximately 5 mm length) from the margin between necrotic and healthy tissue were excised using a sterile scalpel and plated onto potato dextrose agar (PDA; Difco) supplemented with 100 mg/L streptomycin sulfate to suppress fungal contamination. Plates were incubated at 26 ± 2 °C in darkness for 3–5 days. Emerging fungal colonies were purified through hyphal tip transfer to fresh PDA, followed by single-spore isolation to ensure genetic uniformity. For single-spore isolation, conidia were harvested from sporulating cultures, suspended in sterile water, diluted to approximately 10^3^ spores/mL, and 100 μL aliquots were spread onto 2% water agar. After 12–18 h incubation at 26 °C, individual germinating spores were transferred to PDA using a sterile needle under a dissecting microscope. This procedure was repeated twice to establish axenic cultures. Pure cultures were maintained on PDA slants at 4 °C for routine use and preserved in 20% glycerol at −80 °C for long-term storage. From the 30 diseased plants, twenty-three fungal isolates were obtained, showing Fusarium-like colony morphology and conidial structures.

### 4.2. Pathogenicity Testing and Verification of Koch’s Postulates

#### 4.2.1. Inoculum Preparation

Inoculum was prepared using sorghum grain cultures following established methods [42]. Sorghum grains were soaked in distilled water for 12 h, drained, dispensed into 500-mL Erlenmeyer flasks (150 g per flask), and autoclaved twice (121 °C, 30 min) with a 24 h interval between sterilizations. After cooling, grains were inoculated with five 5 mm mycelial plugs from actively growing 5-day-old cultures and incubated at 26 ± 2 °C for 14 days with periodic shaking every 2–3 days to ensure uniform colonization. Colonized grains were air-dried in a laminar flow hood for 24 h before use.

#### 4.2.2. Pathogenicity Assay Design

To compare pathogenicity among the three *Fusarium* species, representative isolates (*F. oxysporum* A3, *F. equiseti* B2, *F. brachygibbosum* A6) were tested, selected for their morphological distinctiveness and clear phylogenetic clustering. Seeds of the susceptible cultivar Dongnong 50 were surface-sterilized (2% NaOCl, 3 min), rinsed thoroughly, and pre-germinated on moist filter paper at 25 °C in darkness for 48 h. For each isolate, colonized sorghum grains (30 g) were thoroughly mixed with 270 g of autoclaved vermiculite (1:9 *w*/*w*) in plastic pots (12 cm diameter × 15 cm height) to achieve uniform distribution of the inoculum. Eight pre-germinated seeds were planted per pot at a depth of 2 cm; after emergence (approximately 7 days), seedlings were thinned to five uniform plants per pot. Non-inoculated controls received an equivalent mixture of autoclaved, non-colonized sorghum grains (30 g) and sterile vermiculite (270 g). The experimental design for each isolate comprised three biological replicates (pots) × five plants per pot × two independent experiments conducted one week apart, totaling 30 plants per isolate. All experiments were performed in a controlled-environment growth chamber (25 ± 2 °C, 16 h photoperiod at 200 μmol m^−2^ s^−1^ photosynthetic photon flux density, 60–70% relative humidity), with pots arranged in a randomized complete block design and rotated daily to minimize positional effects.

#### 4.2.3. Disease Assessment

Disease severity was evaluated at 14 days post-emergence using a modified 0–7 scale adapted from previous *Fusarium* root rot studies [42,43]: 0 = no visible symptoms; 1 = slight root browning affecting <10% of root surface; 3 = blackened main root with lesion expansion covering 30–50% of root system; 5 = severe root rot with >70% root necrosis and stunted shoot growth; 7 = complete root rot with plant death or near-death. The disease severity index (DSI) was calculated as:

DSI = [Σ(number of plants at each grade × grade value)/(total plants assessed × maximum grade)] × 100

Mean DSI values ± standard error were calculated from six replicates (three pots per experiment × two experiments). Isolates were classified as highly pathogenic (DSI ≥ 60), moderately pathogenic (50 ≤ DSI < 60), or weakly pathogenic (DSI < 50) [44]. At 14 dpi, plant height (soil surface to apical meristem), fresh shoot weight, fresh root weight, primary root length, and lateral root number were also measured for each plant. Tissue fresh weights were recorded immediately after excision; root systems were gently washed and blotted dry before measurement.

#### 4.2.4. Fulfillment of Koch’s Postulates

To establish causal relationships between isolated fungi and disease symptoms, Koch’s postulates were rigorously fulfilled for each species. From symptomatic plants in the pathogenicity assays described above, diseased root sections showing characteristic lesions were collected, surface-sterilized, and plated onto PDA with streptomycin. Emerging fungal colonies were purified through single-spore isolation. For each species, three independent re-isolates (one from each biological replicate pot) were confirmed morphologically by examining conidial structures and colony characteristics, then verified molecularly through ITS sequencing. Re-isolated sequences showed ≥99% identity with the original inoculated isolates, confirming that the same fungal species was recovered from diseased tissues and thereby satisfying the fourth postulate and establishing pathogenicity.

### 4.3. Molecular Identification and Phylogenetic Analysis

#### 4.3.1. DNA Extraction and ITS Amplification

Genomic DNA was extracted from fresh mycelium scraped from 7-day-old PDA cultures using the HyperMB Fungal DNA Extraction Kit (Sangon Biotech B690021, Shanghai, China) following the manufacturer’s protocol. DNA concentration was quantified using a NanoDrop 2000 spectrophotometerv1.6.198 (Thermo Fisher Scientific, Waltham, MA, USA), and samples with A_260_/A_280_ ratios between 1.8 and 2.0 were used for PCR. The nuclear ribosomal internal transcribed spacer (ITS) region was amplified using the universal fungal primers ITS1 and ITS4 [45]. PCR reactions (25 μL total volume) contained 12.5 μL 2× Taq Master Mix (Vazyme), 1.0 μL each primer (10 μM), 1.0 μL template DNA (~50 ng), and 9.5 μL sterile deionized water. Thermal cycling conditions were: initial denaturation at 95 °C for 5 min; 35 cycles of 95 °C for 30 s, 55 °C for 30 s, and 72 °C for 1 min; final extension at 72 °C for 10 min; hold at 4 °C. Amplification products were verified by electrophoresis on 1.5% agarose gels stained with GelRed, gel-purified using a Universal DNA Purification Kit (Tiangen DP214, Beijing; China), and sequenced bidirectionally using both PCR primers (Sangon Biotech Co., Ltd., Shanghai, China). Eight representative isolates were sequenced: five isolates representing Type I morphology from all three locations (*F. oxysporum* A1, A3 from Minzhu Town; B4, B7 from Xiangyang Farm; C6 from Changling Lake), two Type II isolates (*F. equiseti* B2 from Xiangyang Farm; C3 from Changling Lake), and one Type III isolate (*F. brachygibbosum* A6 from Minzhu Town). The remaining 15 isolates were assigned to species based on their morphological concordance with the sequenced representatives: 13 additional isolates matched Type I morphology and were identified as *F. oxysporum*, one matched Type II (*F. equiseti*), and one matched Type III (*F. brachygibbosum*).

#### 4.3.2. Sequence Analysis and Phylogenetic Reconstruction

Forward and reverse sequences were assembled and manually edited in BioEdit v7.2.5 (http://www.mbio.ncsu.edu/bioedit (accessed on 22 August 2025)). Consensus sequences were subjected to BLASTn searches against the NCBI GenBank nucleotide database. Species identification was assigned when sequences showed ≥97% identity and ≥94% query coverage to authenticated type or reference strains. All newly generated ITS sequences were deposited in GenBank under accession numbers PQ991234–PQ991241 (Table S2). For phylogenetic analysis, the eight ITS sequences were aligned with closely related reference sequences retrieved from GenBank using MUSCLE implemented in MEGA X v10.2.6 [46]. The alignment was manually inspected and trimmed to 512 bp to remove ambiguous regions. Phylogenetic trees were constructed using two methods: (1) Neighbor-Joining (NJ) with the Kimura 2-parameter nucleotide substitution model, pairwise deletion of gaps, and 1000 bootstrap replicates; and (2) Maximum Likelihood (ML) using RAxML v8.2.12 with the GTR + G model and 1000 rapid bootstrap replicates. *Fusarium sambucinum* (GenBank accession PP874557.1) served as the outgroup. Bootstrap support values ≥ 70% were considered to indicate strong phylogenetic support. Trees were visualized and edited in FigTree v1.4.4.

### 4.4. Germplasm Screening for Disease Resistance

#### 4.4.1. Plant Material and Screening Protocol

A 200 soybean accessions from the germplasm collection of the Heilongjiang Academy of Agricultural Sciences were evaluated, comprising 165 released cultivars and 35 advanced breeding lines, all adapted to the environmental conditions of Northeast China (Table S4). Based on pathogenicity assays, *F. oxysporum* isolate A3 was selected as the challenge pathogen for germplasm screening due to its high aggressiveness (DSI = 68.5). An etiolated seedling hypocotyl inoculation method was employed, optimized for rapid, high-throughput screening [45]. Seeds were size-sorted (6.0–7.5 mm diameter) using graduated sieves to minimize developmental variation, surface-sterilized with 2% sodium hypochlorite for 3 min, rinsed five times with sterile water, and germinated in darkness at 25 ± 1 °C on moist germination paper for 3–4 days until hypocotyls reached 5–7 cm length. Mycelial plugs (15 mm diameter) excised from the actively growing margin of 5-day-old *F. oxysporum* isolate A3cultures were placed directly onto seedling hypocotyls at a position 3–5 cm below the cotyledons. Inoculation sites were wrapped with sterile cotton gauze pre-moistened with sterile distilled water to maintain humidity. Seedlings were bundled in groups of five, loosely wrapped in aluminum foil to maintain darkness and prevent desiccation, and incubated upright in plastic containers under controlled conditions (25 ± 1 °C, continuous darkness for the first 3 days, then 16 h photoperiod, 60–70% RH). Although seedlings were initially etiolated (developed in darkness), subsequent incubation under a photoperiod prevented complete senescence during the 5-day assessment period. The 200 test accessions were divided into 10 experimental batches of 20 accessions each to facilitate manageable screening throughput. Each batch included two check cultivars: Dongnong 50 (susceptible standard) and ‘Tiedou 44’ (resistant standard). For each accession and check, 15 seedlings were tested per experiment (five seedlings per pot, three replicate pots), and the entire experiment was repeated independently, yielding 30 seedlings per accession across both experiments. Non-inoculated controls for selected accessions received sterile, non-colonized PDA plugs (15 mm diameter) placed at identical hypocotyl positions with the same wrapping and incubation procedures to document baseline responses to wounding and environmental conditions.

#### 4.4.2. Disease Evaluation and Resistance Classification

Disease severity was assessed 5 days post-inoculation according to a 0–3 scale (Table 2) adapted from previous studies [47,48]: Grade 0 = no browning, or minimal browning only at the inoculation point; Grade 1 = normal plant and root growth, epidermal browning only, no vascular damage, lesion non-girdling or girdling without vertical extension; Grade 2 = plant stunted, reduced roots, lesion girdling with vertical water-soaked expansion; Grade 3 = complete browning below cotyledons, plant death.

The disease severity index (DSI) was calculated using the formula:

DSI = [Σ(number of plants in each grade × grade value)/(total number of plants × maximum grade)] × 100

For each accession, 15 seedlings were evaluated (five seedlings per replicate × three replicates), and the experiment was repeated twice. Based on average DSI values, accessions were classified as: highly resistant (HR, DSI ≤ 20), resistant (R, 20 < DSI ≤ 40), moderately susceptible (MS, 40 < DSI ≤ 60), susceptible (S, 60 < DSI ≤ 80), or highly susceptible (HS, DSI > 80).

### 4.5. Gene Expression Analysis

#### 4.5.1. Rationale for Gene Selection and Sampling Times

Based on resistance screening results, two genotypes representing phenotypic extremes for molecular characterization were selected: highly resistant H9477F5 (DSI = 15.3 ± 1.8) and highly susceptible HN91 (DSI = 88.7 ± 3.2). Four defense-related genes representing complementary aspects of plant immunity were analyzed: *GmFER* (*Glyma.18G205800*), encoding ferritin that regulates iron homeostasis and limits oxidative damage during pathogen attack [27]; *GmSOD1* (*Glyma.03G242900*), encoding superoxide dismutase that detoxifies reactive oxygen species generated during defense responses [25]; *GmJAZ1* (*Glyma.01G204400*), encoding a jasmonate ZIM-domain protein that represses jasmonic acid signaling, a critical hormone pathway in biotic stress responses [49]; and *GmTAP1* (*Glyma.18G216900*), encoding a histone acetyltransferase previously identified as a susceptibility factor manipulated by oomycete effectors to suppress host immunity [29,30]. These genes integrate oxidative stress management (FER, SOD1), hormone signaling (JAZ1), and chromatin-level transcriptional regulation (TAP1), providing a multi-layered view of defense mechanisms rather than focusing narrowly on a single pathway. Sampling times (1, 3, and 5 days post-inoculation) were selected to capture early (1 dpi), peak (3 dpi), and later (5 dpi) stages of the infection process. Previous studies of soybean-*Fusarium* interactions have shown that 2–4 dpi represents a critical window when defense responses are maximally induced and when resistant versus susceptible phenotypes begin to diverge [50,51]. The 1 dpi time point captures immediate-early responses, 3 dpi captures peak response when pathogen colonization is active, and 5 dpi captures late responses when disease symptoms become visible in susceptible genotypes.

#### 4.5.2. Sample Collection and RNA Extraction

Etiolated seedlings of H9477F5 and HN91 were prepared and inoculated with *F. oxysporum* isolate A3. At 1, 3, and 5 dpi, hypocotyl tissue (approximately 10 mm centered on the inoculation site, including both epidermis and vascular tissue) was excised using a sterile razor blade. Parallel control seedlings received sterile, non-colonized PDA plugs at the same hypocotyl position and were processed identically to account for wounding-induced gene expression and environmental effects. Each biological replicate consisted of hypocotyl tissue pooled from five seedlings; three independent biological replicates were collected per genotype × treatment × time combination. Tissue samples were immediately frozen in liquid nitrogen and stored at −80 °C until processing. Total RNA was extracted using the FastPure Plant Total RNA Isolation Kit (Vazyme RC411-01, Nanjing, China) according to the manufacturer’s instructions, including on-column DNase I treatment to eliminate genomic DNA contamination. RNA quality was assessed by measuring A_260_/A_280_ (acceptable range: 1.8–2.0) and A_260_/A_230_ (>2.0) ratios using a NanoDrop 2000 spectrophotometer (Thermo Fisher Scientific, Waltham, MA, USA). RNA integrity was verified by agarose gel electrophoresis, confirming sharp 28S and 18S ribosomal RNA bands with an approximate 28S:18S intensity ratio of ≥1.5. Only RNA samples meeting all quality criteria were used for cDNA synthesis.

#### 4.5.3. cDNA Synthesis and Quantitative RT-PCR

First-strand cDNA was synthesized from 1 μg total RNA using HiScript III RT SuperMix for qPCR (+gDNA wiper) (Vazyme R323-01) following the manufacturer’s protocol. The resulting cDNA was diluted 1:10 with nuclease-free water and stored at −20 °C. Gene-specific primers for the four target genes and the reference gene *GmActin11* (*Glyma.18G290800*) were designed using Primer3Plus (http://www.primer3plus.com (accessed on 22 Augast 2025)) with the following constraints: amplicon size 100–200 bp, primer length 18–22 nucleotides, melting temperature 58–62 °C, GC content 45–60%, and maximum 3′ self-complementarity ≤ 3. Primer sequences are listed in Table S7. Primer specificity was verified by BLASTn analysis against the soybean genome (*Glycine max* Wm82.a2.v1) and confirmed experimentally by melt-curve analysis, which showed a single peak. Primer amplification efficiency was determined using five-point standard curves constructed from 5-fold serial dilutions of cDNA; all primers exhibited efficiencies between 90% and 110% with R^2^ > 0.99. Quantitative RT-PCR was performed using ChamQ Universal SYBR qPCR Master Mix (Vazyme Q711-02) on a QuantStudio 3 Real-Time PCR System (Applied Biosystems, Foster City, CA, USA). Each 20 μL reaction contained 10 μL SYBR Master Mix, 0.4 μL forward primer (10 μM), 0.4 μL reverse primer (10 μM), 2 μL diluted cDNA template, and 7.2 μL nuclease-free water. Thermal cycling conditions were: initial denaturation at 95 °C for 30 s; 40 cycles of 95 °C for 10 s and 60 °C for 30 s; followed by melt curve analysis from 60 °C to 95 °C (increment 0.5 °C, 5 s per step) to verify single-product amplification. Each biological replicate was analyzed with three technical replicates on the same plate. No-template controls (NTC) and minus-reverse transcriptase controls (–RT) were included on each plate to monitor contamination and genomic DNA carryover. Inter-run calibrator samples (pooled cDNA from all samples) were included across plates to enable normalization of plate-to-plate variation. Relative gene expression was calculated using the 2^(−ΔΔCt) method ]. For each sample, ΔCt was calculated as Ct (target gene) − Ct(*GmActin11*). For each gene and time point, ΔΔCt was calculated as ΔCt(inoculated sample) – ΔCt (mean of genotype-matched non-inoculated controls at 0 dpi), where 0 dpi represents the pre-inoculation baseline sampled immediately before plug application. This normalization approach accounts for any constitutive expression differences between genotypes and isolates, and specifically for fold-change due to *F. oxysporum* inoculation.

#### 4.5.4. Quantification of Fungal Colonization

To confirm successful infection and quantify pathogen biomass in planta, genomic DNA was extracted from the same hypocotyl tissues used for RNA extraction (adjacent 5 mm sections) using a modified cetyltrimethylammonium bromide (CTAB) protocol. Briefly, frozen tissue (100 mg) was ground in liquid nitrogen, suspended in 600 μL preheated (65 °C) CTAB extraction buffer (2% CTAB, 100 mM Tris-HCl pH 8.0, 20 mM EDTA, 1.4 M NaCl, 1% PVP-40), incubated at 65 °C for 30 min with occasional mixing, extracted with chloroform: isoamyl alcohol (24:1), and DNA precipitated with isopropanol. DNA pellets were washed with 70% ethanol, air-dried, and resuspended in 50 μL TE buffer. Fungal biomass was quantified by qPCR targeting the *F. oxysporum* ITS region using species-specific primers Fo-ITS-F and Fo-ITS-R [52]. A standard curve was generated by cloning the *F. oxysporum* ITS amplicon (550 bp) into the pMD19-T vector (Takara), verifying the insert by sequencing, quantifying plasmid DNA concentration, calculating copy number based on molecular weight, and preparing 10-fold serial dilutions ranging from 10^7^ to 10^2^ copies/μL. qPCR was performed as described above using 2 μL of 1:10-diluted gDNA as template. Fungal biomass was expressed as the number of ITS copies per nanogram of total plant DNA. This approach provides absolute quantification of pathogen colonization, enabling comparison of fungal growth rates between resistant and susceptible genotypes.

### 4.6. Statistical Analysis

All statistical analyses were conducted using SPSS Statistics v26.0 (IBM Corp., Armonk, NY, USA) and GraphPad Prism v9.5.1 (GraphPad Software, San Diego, CA, USA). Data were first assessed for normality using the Shapiro–Wilk test and for homogeneity of variance using Levene’s test. When assumptions were satisfied (*p* > 0.05), parametric tests were applied; otherwise, data were log-transformed or analyzed using non-parametric methods. For pathogenicity comparisons among *Fusarium* species and among resistance categories in germplasm screening, DSI data were analyzed by one-way analysis of variance (ANOVA) followed by Tukey’s honest significant difference (HSD) post hoc test for pairwise comparisons (α = 0.05). Reproducibility between the two independent experiments was assessed using Pearson correlation, and data from both experiments were pooled when correlations were high (r > 0.85, *p* < 0.001). Summary statistics for germplasm distribution (mean, median, standard error, skewness, kurtosis) were calculated to characterize the phenotypic variation.

## 5. Conclusions

This study integrated pathogen characterization, germplasm screening, and preliminary molecular analysis to address *Fusarium* root rot in soybean production in Harbin, Northeast China. Through morphological and molecular identification combined with rigorous pathogenicity testing, it was identified that *F. oxysporum* was the dominant and most aggressive pathogen (78% of isolates, DSI = 68.5), with *F. equiseti* (13%, DSI = 52.3) and *F. brachygibbosum* (9%, DSI = 38.7) also present. The first documentation of *F. brachygibbosum* on soybean in Northeast China extends the species’ known geographic range and underscores the need for continued regional pathogen surveillance. Screening of 200 locally adapted germplasm accessions revealed substantial genetic variation for resistance, with 13 highly resistant (6.5%) and 40 resistant (20%) accessions identified. The continuous distribution of disease severity indices (mean = 52.84, range = 0–100) suggests quantitative inheritance involving multiple genes, indicating that breeding strategies should focus on pyramiding moderate-effect resistance alleles from diverse sources rather than relying on single major genes. The identified resistant accessions, particularly varieties with documented resistance to multiple diseases (Tiedou 44, Jiadou 25, Suinong 20), provide practical genetic resources for regional breeding programs, though field validation across multiple environments and years is necessary to confirm resistance stability. Gene expression analysis of two extreme genotypes revealed distinct patterns potentially associated with resistance: elevated expression of oxidative stress response genes (*GmFER, GmSOD1*) and reduced expression of negative regulators (*GmJAZ1, GmTAP1*) in resistant lines compared with susceptible lines. This study provides characterized pathogen isolates for use in resistance screening, resistant germplasm adapted to Northeast China conditions, and preliminary molecular insights that may inform marker development. These resources support ongoing breeding efforts to enhance soybean root rot resistance in this critical production region.

## Figures and Tables

**Table 1:** Species identification, distribution, and pathogenicity of *Fusarium* isolates from soybean root rot in Harbin, Heilongjiang Province.

Species	Number of Isolates (%)	Distribution by Location	DSI ± SE	Pathogenicity Classification
*Fusarium oxysporum*	18 (78%)	MT: 5, XF: 7, CL: 6	68.5 ± 3.2	Highly pathogenic
*Fusarium equiseti*	3 (13%)	XF: 1, CL: 2	52.3 ± 2.8	Moderately pathogenic
*Fusarium brachygibbosum*	2 (9%)	MT: 2	38.7 ± 2.1	Weakly pathogenic

**Table 2:** Disease Severity Grading Criteria for Soybean Root Rot Resistance Screening.

Grade	Symptom Description
0	No browning, or minimal browning only at the inoculation point
1	Normal plant and root growth; epidermal browning only; no vascular damage; lesion non-girdling or girdling without vertical extension
2	Plant stunted; reduced roots; lesion girdling with vertical, water-soaked expansion
3	Complete browning below cotyledons; plant death

## Data Availability

The original data presented in the study are included in the article/Supplementary Materials; further inquiries can be directed to the corresponding authors.

## Supplementary Materials

The following supporting information can be downloaded at: https://www.mdpi.com/article/10.3390/plants15030379/s1, Figure S1: Electrophoresis diagram of PCR products; Figure S2: The ITS sequence comparison chart of *Fusarium oxysporum* on NCBI; Figure S3: The ITS sequence comparison chart of *Fusarium equiseti* on NCBI; Figure S4: The ITS sequence comparison chart of *Fusarium brachygibbosum* on NCBI; Table S1: Morphological characteristics of *Fusarium* species isolated from soybean root rot in Harbin. Table S2: Genbank Login number; Table S3: Morphometric measurements of soybean seedlings 14 days after inoculation with three *Fusarium* species; Table S4: Disease Severity Index Table for 200 Varieties; Table S5: Disease Severity Index Table for pathogenicity identification; Table S6: Disease severity index classification table of 200 materials for resistance; Table S7: Quantitative primers for resistance genes.
