# Supplementary material for: Evaluation of Germplasm Resistance in Several Soybean Accessions Against Soybean Fusarium Root Rot in Harbin, Heilongjiang Province, China
Source: Plants (Basel). 2026 Jan 26;15(3):379. doi: 10.3390/plants15030379 (PMC12899736; doi:10.3390/plants15030379)
Supplement: Supplementary file 1 [file plants-15-00379-s001.zip › plants-4086789-supplementary.pdf]

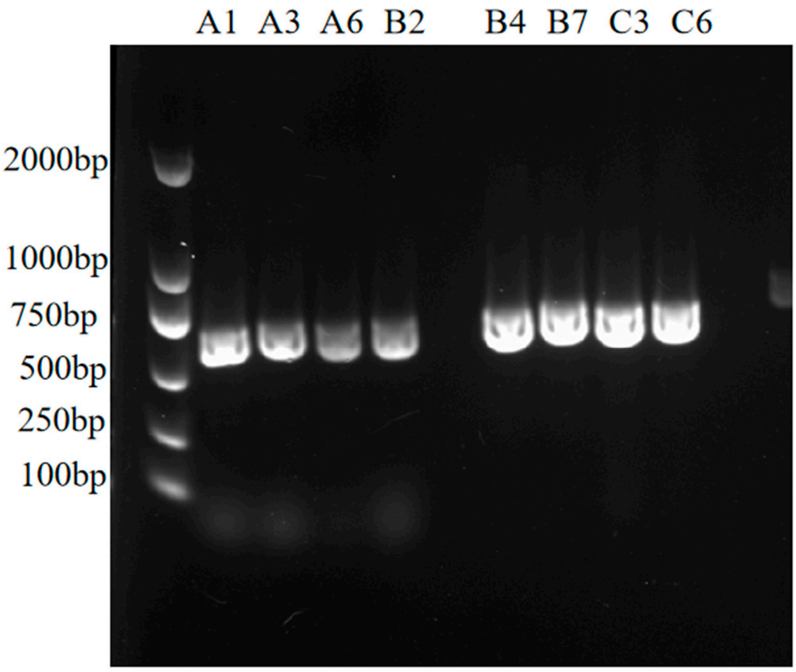

Figure S1. Electrophoresis diagram of PCR products

	Description	Scientific Name	Max Score	Total Score	Query Cover	E value	Per. Ident	Acc. Len	Accession
☑	Fusarium oxysporum isolate LC8-2-1-31 small subunit ribosomal RNA gene, partial sequence; internal transcribed...	Fusarium oxyspo...	1046	1046	100%	0.0	99.65%	572	PP854647.1
☑	Fusarium oxysporum isolate LuC1-1-48 small subunit ribosomal RNA gene, partial sequence; internal transcribed ...	Fusarium oxyspo...	1044	1044	100%	0.0	99.65%	571	PP864100.1
☑	Fusarium oxysporum strain NATG-5 small subunit ribosomal RNA gene, partial sequence; internal transcribed spa...	Fusarium oxyspo...	1044	1044	100%	0.0	99.65%	571	MK673880.1
☑	Fusarium oxysporum isolate LC9-1-8 small subunit ribosomal RNA gene, partial sequence; internal transcribed sp...	Fusarium oxyspo...	1044	1044	100%	0.0	99.65%	573	PQ303237.1
☑	Fusarium inflexum strain YZU 201055 small subunit ribosomal RNA gene, partial sequence; internal transcribed s...	Fusarium inflexum	1044	1044	100%	0.0	99.65%	583	MW855848.[illegible]
☑	Fusarium oxysporum isolate XM14-1 small subunit ribosomal RNA gene, partial sequence; internal transcribed sp...	Fusarium oxyspo...	1044	1044	100%	0.0	99.65%	571	PQ350383.1
☑	Fusarium oxysporum strain CIRM-BRFM 2453 small subunit ribosomal RNA gene, partial sequence; internal trans...	Fusarium oxyspo...	1042	1042	100%	0.0	99.65%	570	PV108972.1
☑	Fusarium sp. strain DNS1-2-1 small subunit ribosomal RNA gene, partial sequence; internal transcribed spacer 1, ...	Fusarium sp.	1042	1042	100%	0.0	99.48%	572	PQ870410.1
☑	Fusarium oxysporum isolate LC1-5-1-8 small subunit ribosomal RNA gene, partial sequence; internal transcribed ...	Fusarium oxyspo...	1038	1038	100%	0.0	99.48%	571	PP838603.1
☑	Fusarium oxysporum isolate LC1-5-1-10 small subunit ribosomal RNA gene, partial sequence; internal transcribed...	Fusarium oxyspo...	1038	1038	100%	0.0	99.48%	571	PP838606.1
☑	Fusarium oxysporum isolate LC1-1-1-51 small subunit ribosomal RNA gene, partial sequence; internal transcribed...	Fusarium oxyspo...	1038	1038	100%	0.0	99.48%	571	PP830108.1
☑	Fusarium oxysporum strain YJTG-6 small subunit ribosomal RNA gene, partial sequence; internal transcribed spa...	Fusarium oxyspo...	1037	1037	100%	0.0	99.47%	574	MK673882.1
☑	Fusarium oxysporum isolate PS5-1-6 small subunit ribosomal RNA gene, partial sequence; internal transcribed sp...	Fusarium oxyspo...	1037	1037	100%	0.0	99.30%	575	PQ303226.1
☑	Fusarium oxysporum isolate C02 small subunit ribosomal RNA gene, partial sequence; internal transcribed spacer...	Fusarium oxyspo...	1037	1037	100%	0.0	99.30%	574	KY910846.1

Figure S2.The ITS sequence comparison chart of *Fusarium oxysporum* on NCBI

☑	Fusarium equiseti isolate SHZ Fe 1 small subunit ribosomal RNA gene, partial sequence; internal transcribed spac…	Fusarium equiseti	987	987	100%	0.0	99.27%	548	MK780235.1
☑	Fusarium equiseti isolate Abr2 3 small subunit ribosomal RNA gene, partial sequence; internal transcribed spacer 1…	Fusarium equiseti	987	987	100%	0.0	99.27%	547	PV186739.1
☑	Fusarium equiseti strain FSK4 small subunit ribosomal RNA gene, partial sequence; internal transcribed spacer 1,…	Fusarium equiseti	987	987	100%	0.0	99.27%	549	OM876894.1
☑	Fusarium equiseti isolate EA1 small subunit ribosomal RNA gene, partial sequence; internal transcribed spacer 1,…	Fusarium equiseti	987	987	100%	0.0	99.27%	547	ON868912.1
☑	Fusarium compactum isolate LJYB01 small subunit ribosomal RNA gene, partial sequence; internal transcribed sp…	Fusarium compa…	985	985	100%	0.0	99.09%	555	OM100556.1
☑	Fusarium equiseti isolate JMF-01 small subunit ribosomal RNA gene, partial sequence; internal transcribed spacer …	Fusarium equiseti	985	985	100%	0.0	99.27%	546	MW404610.
☑	Fusarium equiseti isolate 1247 small subunit ribosomal RNA gene, partial sequence; internal transcribed spacer 1,…	Fusarium equiseti	985	985	99%	0.0	99.27%	546	PP939865.1
☑	Fusarium equiseti isolate JMF-02 small subunit ribosomal RNA gene, partial sequence; internal transcribed spacer …	Fusarium equiseti	985	985	100%	0.0	99.27%	547	MW404611.
☑	Fusarium equiseti isolate LQ144 small subunit ribosomal RNA gene, partial sequence; internal transcribed spacer …	Fusarium equiseti	985	985	100%	0.0	99.27%	548	MK168567.1
☑	Fusarium equiseti isolate 32 small subunit ribosomal RNA gene, partial sequence; internal transcribed spacer 1, 5.…	Fusarium equiseti	985	985	100%	0.0	99.27%	549	KY318493.1
☑	Fusarium equiseti isolate JM2-1 small subunit ribosomal RNA gene, partial sequence; internal transcribed spacer 1…	Fusarium equiseti	983	983	100%	0.0	99.09%	552	PV240289.1
☑	Fusarium equiseti isolate 3 small subunit ribosomal RNA gene, partial sequence; internal transcribed spacer 1, 5.8…	Fusarium equiseti	983	983	100%	0.0	99.09%	572	OR031853.1
☑	Fusarium equiseti isolate B27 small subunit ribosomal RNA gene, partial sequence; internal transcribed spacer 1, 5…	Fusarium equiseti	983	983	99%	0.0	99.27%	545	PQ037764.1
☑	Fusarium equiseti strain 286-09 18S ribosomal RNA gene, partial sequence; internal transcribed spacer 1, 5.8S rib…	Fusarium equiseti	983	983	99%	0.0	99.27%	545	JQ412109.1
☑	Fusarium equiseti isolate ALX53 small subunit ribosomal RNA gene, partial sequence; internal transcribed spacer 1…	Fusarium equiseti	983	983	99%	0.0	99.27%	547	PQ498897.1

Figure S3. The ITS sequence comparison chart of *Fusarium equiseti* on NCBI

☑	Fusarium brachygibbosum strain BMK2 small subunit ribosomal RNA gene, partial sequence; internal transcribed …	Fusarium brachy…	996	996	99%	0.0	99.63%	556	OM876896.1
☑	Fusarium brachygibbosum isolate Fus-C24 small subunit ribosomal RNA gene, partial sequence; internal transcrib…	Fusarium brachy…	996	996	99%	0.0	99.63%	555	PP344799.1
☑	Fusarium brachygibbosum isolate Fus-C24 small subunit ribosomal RNA gene, partial sequence; internal transcrib…	Fusarium brachy…	996	996	99%	0.0	99.63%	553	PP319886.1
☑	Fusarium brachygibbosum strain C 18S ribosomal RNA gene, partial sequence; internal transcribed spacer 1, 5.8…	Fusarium brachy…	996	996	99%	0.0	99.63%	556	KF028369.1
☑	Fusarium brachygibbosum isolate BTJ-2-1 small subunit ribosomal RNA gene, partial sequence; internal transcrib…	Fusarium brachy…	996	996	99%	0.0	99.63%	578	OQ533551.1
☑	Fusarium brachygibbosum isolate KS5 small subunit ribosomal RNA gene, partial sequence; internal transcribed s…	Fusarium brachy…	996	996	99%	0.0	99.63%	557	MW928607.
☑	Fusarium brachygibbosum strain 28 small subunit ribosomal RNA gene, partial sequence; internal transcribed spa…	Fusarium brachy…	994	994	99%	0.0	99.63%	546	PV185829.1
☑	Fusarium oxysporum isolate FP1 small subunit ribosomal RNA gene, partial sequence; internal transcribed spacer…	Fusarium oxyspo…	994	994	99%	0.0	99.63%	555	OQ996845.1
☑	Fusarium brachygibbosum isolate p13-1 small subunit ribosomal RNA gene, partial sequence; internal transcribed…	Fusarium brachy…	994	994	99%	0.0	99.63%	555	MZ026470.1

Figure S4.The ITS sequence comparison chart of *Fusarium brachygibbosum* on NCBI
